# Discovery of Natural Products With Antifungal Potential Through Combinatorial Synergy

**DOI:** 10.3389/fmicb.2022.866840

**Published:** 2022-04-26

**Authors:** Catheryn R. Augostine, Simon V. Avery

**Affiliations:** School of Life Sciences, University of Nottingham, Nottingham, United Kingdom

**Keywords:** drug combinations, fungicide combinations, crop pathogens, fungal pathogens, *Zygosaccharomyces bailii*, *Saccharomyces cerevisiae*

## Abstract

The growing prevalence of antifungal drug resistance coupled with the slow development of new, acceptable drugs and fungicides has raised interest in natural products (NPs) for their therapeutic potential and level of acceptability. However, a number of well-studied NPs are considered promiscuous molecules. In this study, the advantages of drug–drug synergy were exploited for the discovery of pairwise NP combinations with potentiated antifungal activity and, potentially, increased target specificity. A rational approach informed by previously known mechanisms of action of selected NPs did not yield novel antifungal synergies. In contrast, a high-throughput screening approach with yeast revealed 34 potential synergies from 800 combinations of a diverse NP library with four selected NPs of interest (eugenol, EUG; β-escin, ESC; curcumin, CUR; berberine hydrochloride, BER). Dedicated assays validated the most promising synergies, namely, EUG + BER, CUR + sclareol, and BER + pterostilbene (PTE) [fractional inhibitory concentrations (FIC) indices ≤ 0.5 in all cases], reduced to as low as 35 (BER) and 7.9 mg L^–1^ (PTE). These three combinations synergistically inhibited a range of fungi, including human or crop pathogens *Candida albicans*, *Aspergillus fumigatus*, *Zymoseptoria tritici*, and *Botrytis cinerea*, with synergy also against azole-resistant isolates and biofilms. Further investigation indicated roles for mitochondrial membrane depolarization and reactive oxygen species (ROS) formation in the synergistic mechanism of EUG + BER action. This study establishes proof-of-principle for utilizing high-throughput screening of pairwise NP interactions as a tool to find novel antifungal synergies. Such NP synergies, with the potential also for improved specificity, may help in the management of fungal pathogens.

## Introduction

Fungi can have devastating socio-economic impacts through human disease, crop disease, and food spoilage (Brown et al., 2012; Almeida et al., 2019; Avery et al., 2019; Fones et al., 2020). As fungi share many conserved cellular functions with potential host eukaryotes (humans, plants), the discovery of effective antifungal drugs or fungicides is challenging while resistance is growing to existing agents, accentuating the need for discovery of novel measures for fungal control (Roemer and Krysan, 2014; Fisher et al., 2018). On top of this, tightening of regulations and shifting of public attitudes away from the use of traditional chemical actives calls for different approaches to fungal control. Natural products are one group of compounds that are more acceptable in this landscape (Campêlo et al., 2019; Atanasov et al., 2021).

Natural products are increasingly reported in inhibitor discovery programs. Natural products in cancer and infectious disease therapeutics already form the backbone of >50% of drugs being used today, either directly or indirectly (Newman and Cragg, 2016; Rodrigues et al., 2016). Antibiotics are a key example of NP use, including where the antibiotic chemical scaffold has been “copied” from NPs (Wright, 2019). Moreover, the use of NPs in their native form is not to be underestimated considering that they have been moulded throughout evolution to provide benefit to producing organisms, possessing underlying properties required for biological activity (Wright, 2019). Furthermore, regulatory hurdles are commonly lower for NPs. For example, in the application of essential-oil (EO) NPs for food preservation, the European Regulation No. 1334/2008 defines EOs and their active components as flavoring preparations and flavoring substances, respectively. This allows EOs with these properties or which are natural constituents of the product not to be labeled as a preservative, also satisfying “clean label” demand of consumers. Nonetheless, each NP should be judged by its own merits (Debonne et al., 2018; Davies et al., 2021). Despite the numerous advantages of NPs, in recent years several NPs have also been designated promiscuous molecules, having undesirable molecular properties (such as aggregation and membrane perturbation) (Bisson et al., 2016). Related to this, several NPs are very commonly identified as “hits” in high-throughput screens. These NPs have been referred to as pan-assay interference compounds (PAINS) and, in certain cases, invalid metabolic panaceas (IMPs), which are unlikely to progress to lead compounds (Baell and Walters, 2014; Baell, 2016; Bisson et al., 2016). Therefore, enhanced specificity of action is one consideration in the discovery of lead NPs of interest.

Another popular strategy for addressing current challenges of disease- (e.g., fungal) control is the use of drug combinations (Tyers and Wright, 2019). One advantage is that resistance to one drug within the combination may be compensated by the second agent, while rapid pathogen removal can theoretically slow resistance development, where additional mutations would be necessary to overcome the combinatorial inhibition (Spitzer et al., 2017; Tyers and Wright, 2019). Pairs of drug activities may produce additive, antagonistic, or synergistic interactions. Understanding such interactions is important and the potential risk of antagonism between antifungal drugs, for example, has been highlighted (Thomson et al., 2017). Conversely, with drug–drug synergy, the advantages include the use of lower drug doses for effect, so lowering costs and non-specific toxicity concerns (Spitzer et al., 2017). These types of interaction can be distinguished by determination of fractional inhibitory concentration indices (FICIs) (Hsieh et al., 1993). Combinations of certain non-antifungal agents, such as paromomycin and β-escin, were reported to produce marked antifungal synergy against the human pathogen *Candida albicans*, with up to a 64-fold reduction in minimum inhibitory concentrations (MICs) compared with either agent alone (Vallières et al., 2020b). A few synergistic combinations of NPs such as EOs have been described, e.g., a eugenol and thymol combination inhibiting food-borne bacterial pathogens (Liu et al., 2015). Despite the novel activities of interest that have already been uncovered by study of NPs, there remains a diversity of NPs yet to be investigated. In particular, there has yet been very little work dedicated to the discovery of NP–NP synergies. Moreover, the problem of promiscuous activities of some NPs (PAINS, IMPs), discussed above, could potentially be countered with the application of combinational synergy as this should encourage increased potency and specificity (through a common targeted function).

The application of mechanism of action (MOA) knowledge can aid the prediction and discovery of synergistic interactions, as done recently in helping to find novel anti-cancer treatments, for example (Yang et al., 2020). This targeted approach relies on prior knowledge sufficient to enable rational predictions; such as when two agents are known to target similar but not identical processes, which is one basis for synergy (Vallières et al., 2018). On the other hand, the availability of NPs in selective chemical libraries (e.g., libraries which maximize chemical diversity and/or interesting NP activities) facilitates non-targeted screening approaches, including for discovery of lead targets and novel antifungal compounds (Niu and Li, 2019). The use of high-throughput combinatorial screening of chemical-libraries, by combining these with selected compound(s)-of-interest, has recently proved an effective strategy for discovery of novel antifungal synergies (Vallières et al., 2020b). The latter study used standard, non-NP chemical libraries and the approach has not been exploited previously to find NP synergies.

Considering the potential importance of NP discovery for healthcare, food, and agricultural applications, this study tested the hypothesis that either rational or screening-based approaches could be used to find novel, antifungal synergies between NPs. The rational approach capitalized on prior MOA knowledge for three NPs with cell membrane-targeting actions, while wider interrogation utilized an NP-specific chemical library in combination with selected NPs of interest. The study shows the effectiveness of this new screening strategy for finding potent, combinatorial activities among NPs, offering additional tools in the effort to control fungal pathogens.

## Results

### Selected Natural Products With Similar Mechanisms-of-Action Did Not Reveal Combinatorial Synergies

**FIGURE 1:**
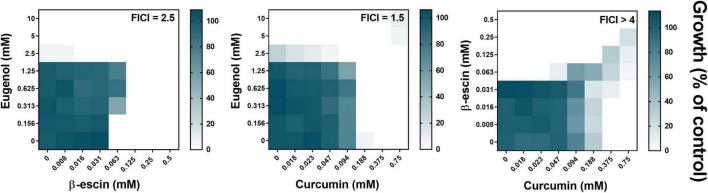
Checkerboard assays of combinatorial growth effects of eugenol, β-escin, and curcumin in *S. cerevisiae*. Assays were performed according to the EUCAST procedure in YPD broth with *S. cerevisiae* W303 at the indicated concentrations of eugenol, β-escin, and curcumin. Growth values (scale to the right) represent means from three independent experiments, calculated as percentages of growth (OD_600_) with the NPs relative to the minus-NP control. FICI, fractional inhibitory concentration index, calculated from data after 24 h growth at 30°C; growth values < 5% were assigned as no-growth (Hsieh et al., 1993). Corresponding data for *S. cerevisiae* BY4743 are shown in Supplementary Figure 1.

It was hypothesized that the natural products eugenol (EUG), β-escin, and curcumin (CUR) may act synergistically in combination. This was based on their related, reported mechanisms of action: in causing lipid peroxidation and disruption of cell membrane integrity (EUG), pore formation within the cell membrane (β-escin), and interactions with ABC drug transporters and *ERG3* gene downregulation, leading to decreased membrane ergosterol and permeability (CUR) (Morton and Main, 2013; Moghadamtousi et al., 2014; Marchese et al., 2017). Checkerboard assays measuring growth of the model yeast *Saccharomyces cerevisiae* were used to assess synergy. These showed that pairwise combinations of EUG, β-escin, or CUR did not present any synergistic interaction: all three of the fractional inhibitory concentration (FIC) index values for these pairs of NPs were > 0.5 (Figure 1). In fact, both the EUG + β-escin and CUR + β-escin combinations exhibited antagonism (with FIC indices ≥ 2.5). The interaction between EUG and CUR was deemed additive (FIC index, 1.5). These observations were reproduced in two common laboratory strains of *S. cerevisiae*, W303 (Figure 1) and BY4743 (Supplementary Figure 1). The results did not support the starting hypothesis.

**FIGURE 2:**
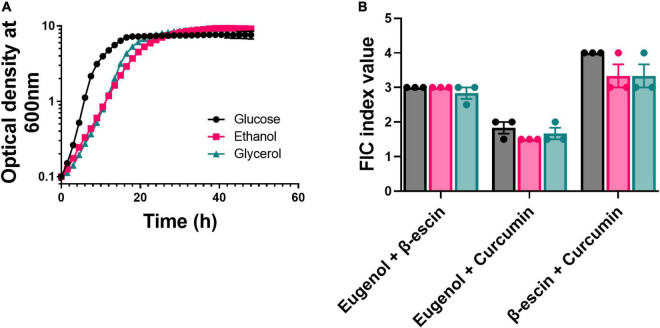
Effect of metabolic environment on the stability of combinatorial interactions. **(A)** Growth curves for *S. cerevisiae* W303 in YEP medium supplemented with either glucose, glycerol, or ethanol (all at 2% w/v). Each point represents the mean of three independent experiments ± SEM (error bars did not exceed the dimensions of the symbols). **(B)** *S. cerevisiae* W303 was treated in checkerboard format with combinations of eugenol, β-escin, and curcumin in YEP supplemented with 2% either glucose (black), glycerol (pink), or ethanol (green). Percentage growth was used to calculate FIC indices. Each bar represents the mean of three independent experiments ± SEM. The corresponding checkerboard data are presented in Supplementary Figure 2.

An organism’s microenvironment can be an important determinant of drug–drug interaction, with previous work reporting a shift from antagonism to synergism for certain antibiotic combinations when bacteria were incubated with glycerol or ethanol instead of glucose (Cokol et al., 2018). To test for similar effects on the stability of the combinatorial interactions among EUG, β-escin, and CUR, these were compared during yeast growth in different carbon sources. [These experiments focused on strain W303, as strain BY4743 has defects in the regulation of respiratory genes (Hon et al., 2005), affecting growth on respired substrates like glycerol and ethanol.] Growth of *S. cerevisiae* W303 was only slightly slower on the respiratory substrates than on glucose, which is fermentable (Figure 2A). Moreover, the antagonistic and additive combinatorial effects observed in glucose (Figure 1), were reproduced in the other carbon sources (Figure 2B and Supplementary Figure 2). The results indicate, for these combinations, that the carbon source and fermentative or respiratory metabolism were not important determinants of these NP–NP interactions.

### Screen of Natural Product Library in Combinations With Selected Natural Products Reveals Multiple Combinatorial Antifungal Candidates

**FIGURE 3:**
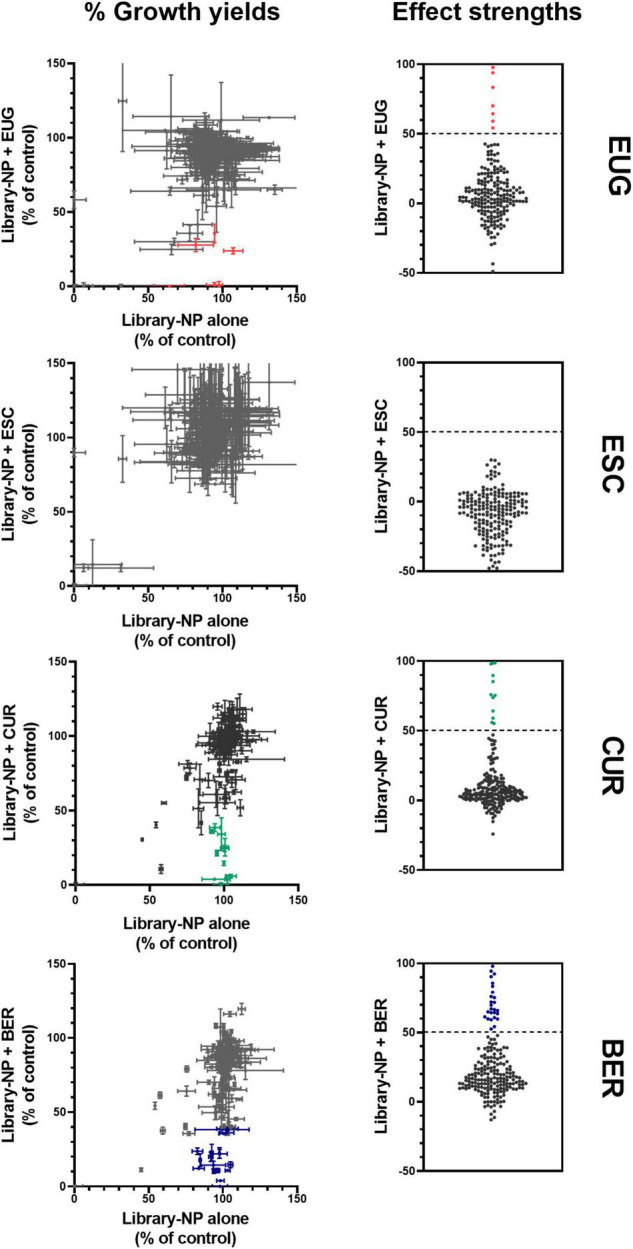
A screen of library NPs in combinations with selected NPs of interest, against the growth of *S. cerevisiae.* Left: normalized growth of *S. cerevisiae* W303 was calculated from OD_600_ values after 24 h with and without NPs, for each of the library NPs both in the absence (*x*-axis) or in the presence (*y*-axis) of 750 μM eugenol (EUG), 12.5 μM β-escin (ESC), 50 μM curcumin (CUR), or 350 μM berberine (BER). Each point represents the mean ± SEM calculated from two independent experiments. Right: effect strengths were determined from [(% growth with library agent) - (% growth with library agent + second agent)] for the different combinations; color is used to highlight those with an effect strength > 50. The underlying data for each combination from the screen are listed in Supplementary Table 1.

To widen the net for enabling the discovery of novel NP antifungal synergies, the Puretitre NP compound library^1^ was screened in combinations with the NPs of interest EUG, CUR, and β-escin. The Puretitre library was selected as the 200 NP compounds it comprises mostly have described use in traditional medicine, possessing both high bioactivity and relatively low toxicity so enhancing translational potential. An additional screen of the library in combination with the NP berberine (BER) is described, as BER had been identified as a hit compound in an initial screen of the library with EUG (described later), and there are previous reports of antimicrobial BER actions (Xu et al., 2009; Dhamgaye et al., 2014; Liu et al., 2015). For the combinatorial screening tests, the four selected NPs were supplied at concentrations that were very near sub-inhibitory (∼10% reduction of growth yield compared to no-drug control, see Supplementary Figure 3), to maximize sensitivity-of-detection of novel synergistic interactions. The library drugs were supplied at a largely sub-inhibitory concentration (100 μM) suggested by the manufacturer and consistent with similar previous work (Vallières et al., 2020b). The screens were performed in *S. cerevisiae* to maximize chances of identifying broad-spectrum antifungal hit combinations, as pathogens commonly have higher intrinsic resistance so offering lower sensitivity of detection of potential synergies. In total, screening of 800 different NP combinations in *S. cerevisiae* revealed 34 candidate interactions of interest (Figure 3 and Supplementary Table 1): these potential synergies (highlighted in color, Figure 3) were identified according to calculated effect strength > 50 (Vallières et al., 2018, 2020a), from the comparison of growth in the presence of each library compound either with or without the second NP (see Section “Materials and Methods”). The screens with EUG revealed six potentially synergistic interactions and with CUR, 11 hits, while no interactions of interest were found with β-escin (Figure 3). As mentioned earlier, one of the strongest interactions with EUG was with library-compound BER (detailed later) and a subsequent screen of the library in combination with BER suggested 17 potential synergies.

### Corroboration of Candidate Synergies and Activity Against Fungal Pathogens

**FIGURE 4:**
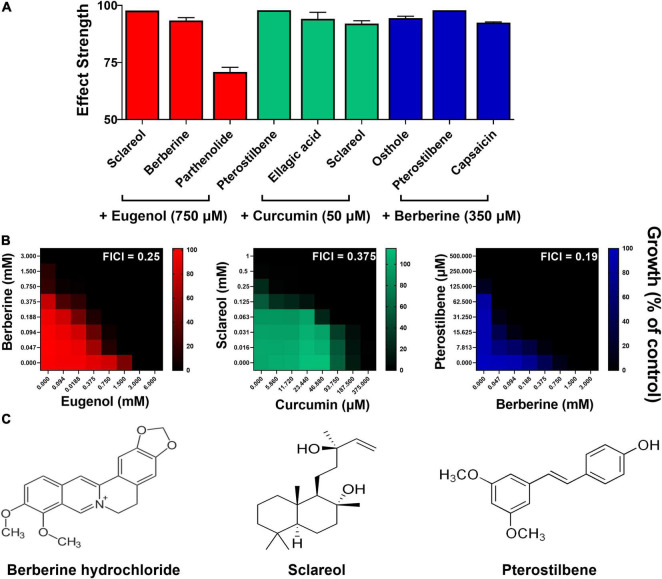
Corroboration of synergies in *S. cerevisiae*. **(A)** Mean effect strengths ± SEM from each screen (Figure 3) for selected combinations of interest (*n* = 2). **(B)** Checkerboard assays of combinatorial growth effects performed according to the EUCAST procedure in YPD broth with *S. cerevisiae* W303 at the indicated concentrations of eugenol, curcumin, berberine, sclareol, and pterostilbene. The growth values represent the mean of three independent experiments calculated as percentages of growth (OD_600_) with the natural products relative to the minus-NP control, after 24 h growth at 30°C. FICI, fractional inhibitory concentration index, calculated from the data and where growth < 5% of the control was assigned as no-growth (Hsieh et al., 1993). **(C)** Chemical structures of library compounds giving the strongest effect strengths from each screen and validated in panel **(B)**. Checkerboard data for additional combinations are presented in Supplementary Figure 4.

To corroborate synergistic interactions from the most promising screen combinations, checkerboard analysis was performed initially in *S. cerevisiae*. The three combinations with the greatest effect strengths from each of the three screens that indicated synergies were selected (Figure 4A). Synergy was corroborated for all nine of these combinations by checkerboard analysis (Supplementary Figure 4). The most promising checkerboard result for each of eugenol, curcumin, and berberine is displayed in Figure 4B, with the corresponding library-compound structures in Figure 4C. The other tested combinations gave FIC index values between 0.25 and ≤0.5 (Supplementary Figure 4) but were not pursued further in this study. For eugenol, the EUG + BER combination reduced the MICs for both compounds by up to 8-fold (Figure 4B). Checkerboard analysis for CUR with sclareol also indicated strong synergy, with MICs reduced by up to 4- and 8-fold, respectively, while the combination of BER with pterostilbene reduced these agents’ MICs by up to 16- and 8-fold, respectively.

To assess the wider efficacies of the three selected combinations, they were tested against fungi that are either important human pathogens, including drug-resistant isolates (*Candida albicans*, *C. glabrata*, *A. fumigatus*); phytopathogens (*B. cinerea*, *Z. tritici*); or a food spoilage organism (*Zygosaccharomyces bailli*). The combinatorial effects determined from checkerboard analysis with this wider range of fungi revealed synergy in all cases, with FIC index values ranging between 0.19 and 0.5, the lowest values suggesting the strongest synergy was with EUG + BER (Table 1). Synergies were also retained in drug- (azole-) resistant isolates of *C. albicans* and *A. fumigatus.* In addition, the EUG + BER combination retained synergistic activity against biofilms of *C. albicans*, showing a significantly greater observed effect of the combination than would be expected from an additive interaction of the observed individual-NP effects (Supplementary Figure 5A). This evidence supported our initial use of NP combinatorial screening (with *S. cerevisiae*) to find novel NP synergies that had broad antifungal spectra.

**TABLE 1:** Values for FIC index for selected NP combinations against diverse human-, plant-pathogenic, and spoilage fungi.

Organism	Eugenol + Berberine	Curcumin + Sclareol	Berberine + Pterostilbene
*C. albicans*	0.50^a^	≤0.50	≤0.38^b^
*C. albicans (azole*^R^)	0.50	>0.50	≤0.50
*C. glabrata*	0.19	≤0.38	≤0.50
*Z. tritici*	0.19	≤0.25	≤0.38
*B. cinerea*	0.25	≤0.25	0.26
*A. fumigatus*	0.25	≤0.25	0.50
*A. fumigatus (azole*^R^)	0.19	≤0.50	0.38
*Z. bailli*	0.31	≤0.50	0.38

*^a^FIC index values were determined by checkerboard analysis with the indicated fungi.*

*^b^≤symbol indicates that the calculated FICI represents the highest possible FIC value for the combination; as inhibitory concentrations were not achieved at the highest doses used in checkerboards, precluding absolute MIC definition for certain agents when supplied individually.*

### The Berberine + Eugenol Combination Causes Synergistic Depolarization of the Mitochondrial Membrane

**FIGURE 5:**
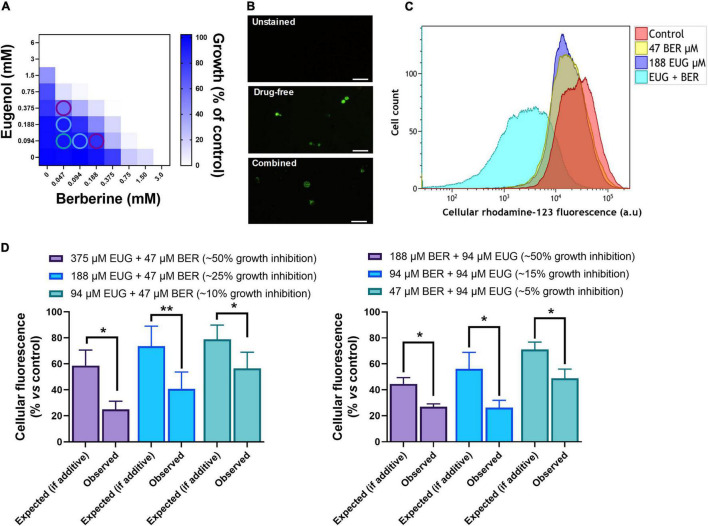
Mitochondrial membrane depolarization in eugenol- and berberine-treated yeast cells. **(A)** Checkerboard assays of combinatorial growth effects were performed as described in Figures 1, 4. Combination concentrations that were subsequently tested for mitochondrial membrane depolarization are circled. **(B)** Microscopic imaging of *S. cerevisiae* cells treated with or without 94 μM EUG and 188 μM BER for 24 h, stained with rhodamine-123. Images were captured using a ×40 objective lens, through a FITC-filter; scale bar, 20 μm. Images are representative of three biological replicates. **(C)** Flow cytometric histograms for cells incubated for 24 h without (control) or with the indicated concentrations of EUG and BER. Cells were then stained with rhodamine 123 before analysis of fluorescence; a.u., arbitrary units. **(D)** Observed effects of combinations were obtained experimentally from median fluorescence of rhodamine 123-stained cells exposed to the EUG + BER combination [derived from corresponding flow cytometric data as in panel **(B)**], normalized to the no drug control (100%). Expected effects were calculated by multiplication of the % median-fluorescence determinations obtained for the corresponding individual-compound effects. Values represent means ± SEM from four independent experiments: *p < 0.05 and **p < 0.01 according to paired *t*-tests. EUG, eugenol; BER, berberine.

As the BER + EUG combination gave the strongest overall synergy it was chosen for further investigation of the mechanism of synergistic action. We hypothesized that the synergy between these agents was centered on mitochondria as a target, as previous studies have indicated that each agent causes mitochondrial perturbation [and associated reactive oxygen species (ROS) production] as a mechanism of cell inhibition (Dhamgaye et al., 2014; Alves et al., 2017). Initially, a rho^0^ mutant was tested to assess whether the loss of mitochondrial DNA (mtDNA) could rescue the synergy between BER and EUG. Synergy was retained in the rho^0^ mutant but was weakened with a FIC index of 0.47 compared with 0.25 in the parental wild type (*p* = 0.028) (Supplementary Figure 6). As the result supported some role for mitochondria in the synergy, we focused next on specific mitochondrial functions. Mitochondrial membrane depolarization by both EUG and BER, individually, has been reported previously (da Silva et al., 2016; Alves et al., 2017). Furthermore, polarity is partly retained in the absence of mtDNA (Appleby et al., 1999; Vowinckel et al., 2021), consistent with the partial-retention of synergy in the rho^0^ mutant shown above. Therefore, we probed mitochondrial membrane depolarization (MMD), using flow cytometric analysis of decreased retention of rhodamine 123 fluorescence by cells, at doses of EUG + BER combinations giving approximately 10, 25, and 50% growth inhibition (Figure 5A). Microscopic observation confirmed cell staining with rhodamine-123 and suggested decreased fluorescence in cells treated with EUG + BER (Figure 5B). The flow cytometric data (presented for the intermediate dosage condition in Figure 5C) corroborated that either NP alone produced decreases in rhodamine 123 stainings. Moreover, the effect was markedly stronger when the NPs were combined. To indicate whether combinatorial effects on retention of the reporter were additive or synergistic, the data were normalized to median fluorescence as percentages of the no-drug controls (see Section “Materials and Methods”). This enabled comparison of the outcome to be “expected” if the combinatorial effect was additive—calculated by multiplying the two fluorescence-medians (% vs. control) obtained for each NP individually—with the observed outcome for the combination (Figure 5D). In all combination concentrations tested (Figure 5A), the observed effects were significantly greater than those which would be expected from additive interactions, suggesting that EUG + BER caused synergistic MMD (Figure 5D). A similar experiment was performed with *C. albicans*, and showed synergistic MMD also for this yeast pathogen (at a combination concentration giving ∼40% growth inhibition) (Supplementary Figures 5B–D). As effects were evident at combination doses including those near sub-inhibitory to growth, it implied that synergistic MMD was not a non-specific consequence of synergistic growth inhibition but consistent with a mechanistic role for MMD in the antifungal synergy between EUG and BER.

### Action of Mitochondrial Reactive Oxygen Species in the Synergy Between Eugenol and Berberine

**FIGURE 6:**
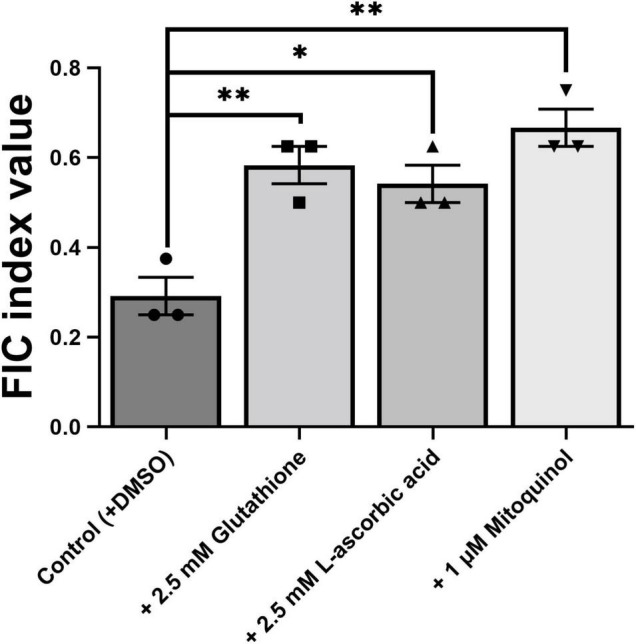
Effect of antioxidants on the eugenol plus berberine synergy. Values plotted to represent mean FICI values from three independent checkerboard experiments performed with *S. cerevisiae* W303, as described in Figures 1, 4, with the inclusion of the antioxidants at the specified concentrations. Bar heights show means ± SEM. *p < 0.05 and **p < 0.05, according to unpaired *t*-tests. The relevant checkerboard data are presented in Supplementary Figure 6.

To further elucidate the mechanism of EUG + BER synergy, we focused on the reported effects of ROS in the individual actions of both these agents (Khan et al., 2011; Dhamgaye et al., 2014). To do this we chose to make use of antioxidant molecules and yeast gene deletants with well-characterized properties, rather than fluorescent ROS probes which can be less specific (Winterbourn, 2014). First, the effects of added antioxidants on the synergy were evaluated. Both glutathione and L-ascorbic acid significantly increased the FIC index value, beyond the threshold (≤0.5) that normally encompasses synergy (Figure 6; checkerboard data are shown in Supplementary Figure 7). A similar effect was also evident for the mitochondria-targeted antioxidant, mitoquinol, where suppression of synergy appeared to be slightly stronger than for glutathione and L-ascorbic acid. The results supported the hypothesis that ROS are important for establishing the synergy between EUG + BER.

**FIGURE 7:**
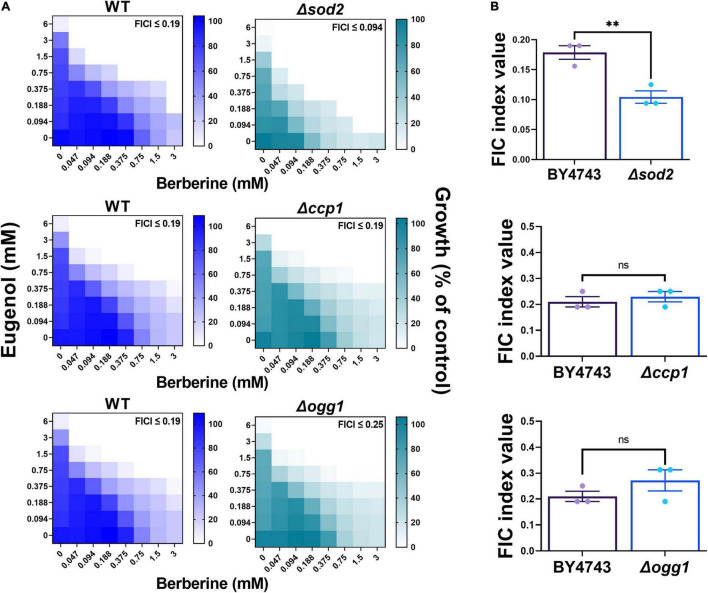
Eugenol plus berberine synergy in deletion strains defective for mitochondrial antioxidant proteins. **(A)** Checkerboard assays of combinatorial growth effects performed according to the EUCAST procedure in YPD broth within *S. cerevisiae* BY4743 (WT) and isogenic deletion mutants Δ*sod2*, Δ*ccp1*, and Δ*ogg1* at the indicated concentrations of eugenol and berberine. The growth values represent the mean of three independent experiments calculated as percentages of growth (OD_600_) with the natural products relative to the minus-NP control, after 24 h at 30°C. FICI, fractional inhibitory concentration index, calculated from the data and where growth < 5% of the control was assigned as no-growth (Hsieh et al., 1993). **(B)** FIC indices are plotted from three independent checkerboard experiments, with bar-height showing mean ± SEM. **p < 0.01, unpaired *t*-test. ns, not significant.

In keeping with the earlier depolarization results for mitochondrial membrane function, and the indication that (mitochondrial) ROS production is important for the synergy, next we focused specifically on the involvement of mitochondrial ROS in the EUG + BER interaction. Deletion mutants defective for three mitochondrial antioxidant functions were tested: Δ*sod2* cells lacking the mitochondrial superoxide dismutase that scavenges mitochondrial superoxide; Δ*ogg1* cells lacking a DNA glycosylase that protects the mitochondrial genome from oxidative damage; and Δ*ccp1* cells lacking the mitochondrial cytochrome C peroxidase. The Δ*ogg1* and Δ*ccp1* deletants exhibited FIC indices with EUG + BER that were not significantly different from that of the wild type (Figure 7). In contrast, synergy was accentuated in cells defective for Sod2p, reflected by an FIC index in the mutant that was ∼50% of that in the wild type (Figure 7). This evidence for partial suppression of the synergy by Sod2p (in wild-type cells) further suggests that ROS, more specifically, mitochondrial superoxide is important for the mechanism of synergistic eugenol–berberine action.

## Discussion

This study highlights the potential value of using NP combinations for fungal control, and how a high-throughput screening approach can enable the ready discovery of novel NP synergies. The high-throughput approach proved more successful here for that purpose, compared with a rational approach based on prior mechanism-of-action knowledge. NPs have been the focus of considerable recent attention for finding novel fungal inhibitors, as concern has grown over diminishing treatment options in the face of growing resistance to existing agents (Aldholmi et al., 2019) or toxicity concerns, including environmental. This work identifies several potential lead NP synergies arising from the pairwise NP screening procedure, which display broad-spectrum inhibition of diverse pathogenic or spoilage fungi. This spectrum of fungi suggests potential for the approach to yield effective NP treatment options in crop protection, human health, or food preservation; noting of course that each of these presents additional sector-specific hurdles including safety and regulatory.

High-throughput screening of individual NPs has been historically important for identifying novel compounds of interest, including in the treatment of cancer, infectious disease, cardiovascular disease, and inflammation (Waltenberger et al., 2016; Attiq et al., 2018; Newman and Cragg, 2020). Moreover, certain NPs have been quite well-characterized, and this information can feed into the development of selective NP chemical libraries (Wright, 2019). The four compounds chosen here for pairwise screens against the NP library had all been reported previously to exhibit at least some mild antifungal activity. One advantage of combinatorial treatments is in enabling the use of lower dosages of both agents—especially when there is synergy—without compromising antifungal efficacy (Vallières et al., 2018). This can be particularly important for NPs, where bioactivity of the NP alone is commonly insufficient (Atanasov et al., 2021). Another drawback of NPs is that often little is known about their mode of action, although this can, of course, be addressed with particular NPs of interest that attract research attention. The NPs used in this study were all selected (by us and the NP library compilers) based on available prior knowledge, including mechanisms of action (MOAs). This work has highlighted that commonalities in MOA between compounds, such as the reported membrane targeting actions of the three NPs initially examined here, eugenol, β-escin, and curcumin, does not assure synergies when applied in combinations and can even produce antagonism between them. These combinations were also not influenced by the carbon source, being stable across respiratory and fermentative growth conditions. Nevertheless, prior knowledge is an advantage for elucidating MOAs underlying any novel synergies between NP pairs, as done here in substantiating that the production of ROS and mitochondrial-membrane depolarization appear to be important for the eugenol/berberine antifungal synergy. It is important to note that such modes of synergistic action are not necessarily the same as the primary, individual-agent MOAs, considering that synergy involves a gain of (inhibitory) activity (Vallières et al., 2020b). The gain of inhibitory action during synergy may be important for countering the challenge of promiscuous activities, associated with many studied NPs (Baell, 2016). This gain of action adds specificity, as synergy typically centers on a common target of the individual agents. For potential translational applications, it is important to guard against promiscuity of action in routine NP-screening studies (Baell, 2016; Bisson et al., 2016). The exploitation of synergy offers a route to help address that.

Among the 34 candidate synergies identified here from 800 interactions tested, nine were chosen for validation and from these, three were taken further for additional investigation. It is encouraging that only a limited number of candidates were identified from the screen, as it supports the notion that assay for synergy should restrict the scope for promiscuity compared with individual-NP activities. The BER + pterostilbene (PTE) combination produced up to 16-fold (BER) and 8-fold (PTE) reductions in MICs for inhibition of *S. cerevisiae* and the synergy was retained against fungal pathogens. Individually, BER and PTE have previously reported activities against *C. albicans*, with reported MOAs linked to the Ras/cyclic AMP pathway (PTE) or membrane damage (BER) (Li et al., 2014; da Silva et al., 2016). The CUR + SCL combination gave similar synergistic potency and spectrum as BER + PTE. Previously, CUR has been shown to downregulate the *ERG3* gene leading to decreased plasma-membrane ergosterol and accumulation of inhibitory precursors in ergosterol biosynthesis, ROS, and cell death (Sharma et al., 2012). Whereas, SCL was reported to induce uncoupling of mitochondrial oxidative phosphorylation in *B. cinerea* (Mendoza et al., 2015). Accordingly, one possible explanation for the observed synergy could be centered on interaction at the mitochondria between the uncoupling, membrane perturbation, and/or ROS actions of the two drugs. Synergy commonly arises between agents that perturb a common process, potentially through a different molecular target (Vallières et al., 2018).

To test a synergistic MOA experimentally, here we concentrated on EUG + BER as this combination gave the lowest FIC index value (strongest synergy) against several of the fungal pathogens tested. Knowledge of previous indications of commonalities in fungus-inhibitory actions of EUG and BER (He et al., 2007; Darvishi et al., 2013; Dhamgaye et al., 2014; da Silva et al., 2016; Das et al., 2016; Marchese et al., 2017) informed the consequent demonstration that the combined agents provoked a synergistic depolarization of the mitochondrial membrane, that the synergy is significantly suppressed by the addition of antioxidants and that the synergy was accentuated in cells defective for Sod2-mediated mitochondrial ROS scavenging. Whereas the focus of this study was on NP synergies, the results do suggest the possibility of synergistic interactions with pro-oxidant molecules such as H_2_O_2_ or superoxide-generating compounds. Taking the evidence together, a model for the synergistic MOA can be proposed whereby synergistic depolarization of the mitochondrial membrane potential is associated with mitochondrial dysfunction and increased mitochondrial ROS production, culminating in accentuated cellular stress and growth inhibition that is greater than a sum of the individual-NP effects.

Concerning the potential for application, one consideration is cost. Berberine and eugenol, for example, are relatively inexpensive. These are purchasable from research suppliers for less than ∼10 USD per gram, whereas the equivalent pricing for the common antifungal drug amphotericin B is ∼1,000 USD per gram. Regarding safety, eugenol is already used in pharmaceutical products (commonly as a local antiseptic and analgesic), as a food preservative, in agriculture (pest control) and in cosmetics and can be administered at 2.5 mg/kg body weight per day in humans (FAO/WHO, 1982; Ulanowska and Olas, 2021). Similarly, numerous studies have supported the application of berberine for therapeutic purposes in humans in relation to different conditions (diabetes, anti-cancer, anti-inflammation, antioxidant, and cardiovascular effects), owing partly to favourable absorption and toxicity properties (Neag et al., 2018; Song et al., 2020). Clinical trials using berberine have used dosing at between 1 and 2 g per day, a level that was deemed safe for a period of at least 6 months (Zeng et al., 2003). In this study, the effective concentrations of these particular agents in combination were up to a maximum of 279 mg L^–1^ BER and 123 mg L^–1^ EUG (calculated from the most resistant pathogen tested, *Candida albicans*). However, it is important to highlight that the effective concentrations could be quite different *in vivo* or with synergistic combinations of more-potent NPs. Accordingly, before any possible commercial or clinical use of novel drug combinations, dosaging needs to be evaluated *in vivo* as a further criterion alongside registration and other regulatory hurdles. For the purposes of this study, the focus was primarily on establishing proof-of-principle, i.e., the demonstration of scope for *in vitro* discovery of antifungal synergies among NP combinations.

Importantly, the lead combinations from this study synergistically inhibited a range of important pathogenic and spoilage ascomycete fungi, including activity against *C. albicans* biofilms (EUG + BER). Synergy was also retained in azole-resistant isolates of *C. albicans* and *A. fumigatus*, suggesting additional potential relevance for the clinical setting where azoles are key drugs for treating invasive infection (Ben-Ami, 2018; Jenks and Hoenigl, 2018). Both NPs are also reported to synergize with fluconazole against azole-resistant *C. albicans* (Quan et al., 2006; Ahmad et al., 2010) enhancing potential scopes of clinical applications. Additional future work may extend tests to important emerging pathogens such as *C. auris*. Despite its potential, the strategy of using synergistic combinations must be carefully monitored and controlled, as resistance development to one agent is likely to abolish pathogen inhibition, given that the second compound may be supplied at too low a concentration to exert inhibition (Davies et al., 2021). Nevertheless, the limited range of effective drugs available for control of fungal pathogens emphasizes the desirability of effective alternative options, such as that offered by combinatorial synergy. By showing that this principle can be extended successfully to NPs, this study promises a potential treasure-trove of novel synergistic interactions ripe for discovery among the diverse plant extracts and other NP sources and libraries available worldwide, beyond the potential combinations of interest revealed here.

## Materials and Methods

### Strains, Culture, and Maintenance

The principal yeast strain backgrounds were *S. cerevisiae* W303 (*MATa/MAT*α *leu2-3,112 trp1-1 can1-100 ura3-1 ade2-1 his3-11,15 [phi*+*]*) and BY4743 (*MATa/MAT*α *his3-1/his3-1 leu2-0/leu2-0 met15-0/MET15 LYS2/lys2-0 ura3-0/ura3-0*). A rho^0^ mutant was derived from *S. cerevisiae* BY4743 by sub-culturing three times on YPD agar supplemented with 40 μg mL^–1^ ethidium bromide. It was confirmed that the resultant mutant was defective for respiratory growth by a lack of growth on YPG agar (recipe as for YPD agar, below, but with glycerol replacing glucose). The *sod2*Δ, *ogg1*Δ, and *ccp1*Δ homozygous diploid deletants (obtained from Euroscarf, Germany), were in the BY4743 background. Other yeast species used in this study included *Z. bailli* strain NCYC1766, *Candida glabrata* BG2, *Candida albicans* SC5314, and an azole-resistant isolate, *C. albicans* J942148, kindly provided by Carol Munro and Donna MacCallum (University of Aberdeen, United Kingdom). Yeasts were maintained and grown at 30°C (*S. cerevisiae*) or 37°C (*C. albicans* and *C. glabrata*) in YPD broth [2% peptone (Oxoid, Basingstoke, United Kingdom), 1% yeast extract (Oxoid), 2% D-glucose]. For experimental purposes, the yeasts were streaked onto YPD agar [recipe as for YPD broth but with the inclusion of 1.5% agar] from −80°C glycerol stocks and cultured for at least 48 h before single colonies were picked for sub-culture to the broth as described below.

Filamentous fungi used in the study were *A. fumigatus* CBS 144.89 and an azole-resistant isolate *A. fumigatus* 3216 (kindly provided by Matthias Brock, University of Nottingham, United Kingdom), *Z. tritici* K4418 (kindly provided by Syngenta, United Kingdom) and *B. cinerea* SAR109940. The filamentous fungi were routinely maintained and grown either on Aspergillus complete medium (ACM) at 37°C for *A. fumigatus*, or Potato Dextrose Agar (PDA, Oxoid) or Potato Dextrose Broth (PDB, Sigma) at room temperature for *Z. tritici* and *B. cinerea* (Vallières et al., 2018). Strains of *A. fumigatus* from −80°C glycerol stocks were grown on PDA slopes for 72 h at 37°C before spores were then harvested for use in experiments. *Z. tritici* and *B. cinerea* were cultured for 7 days from −80°C glycerol stocks prior to harvesting pycnidia for experimental use. Where necessary, media were solidified with 1.5% agar (Sigma, United Kingdom).

### Natural Product and Antioxidant Chemicals

Eugenol, β-escin, curcumin, berberine hydrochloride, sclareol, capsaicin, parthenolide, ellagic acid, glutathione, L-ascorbic acid were from Sigma–Aldrich (United Kingdom); osthole and pterostilbene were from Stratech (United Kingdom) and mitoquinol from Cayman Chemical Company (United Kingdom); all other NPs were components of the Puretitre natural compound library from Caithness Biotechnologies (United Kingdom). All of the above except eugenol (70% ethanol), glutathione, and L-ascorbic acid (dH_2_O) were dissolved in dimethyl sulfoxide (DMSO, Sigma United Kingdom) and added to growth media from the following stock solutions prepared in those solvents: eugenol, 500 mM; glutathione, 375 mM, L-ascorbic acid, 500 mM; osthole, 200 mM; pterostilbene, 200 mM; β-escin, 50 mM; curcumin, 50 mM; berberine hydrochloride, 200 mM; sclareol, 130 mM; capsaicin, 200 mM; parthenolide, 130 mM; ellagic acid, 33.3 mM, mitoquinol, 2.94 mM.

### Checkerboard Assays and Other Growth Inhibition Assays

All culturing and preparation for checkerboard assays adhered to EUCAST guidelines, except for the use of YPD broth, ACM, or PDB instead of RPMI as medium (Arendrup et al., 2012). Briefly, for yeasts, overnight cultures in YPD broth, 120 rev. min^–1^, 30°C or 37°C (see above), derived from single colonies, were diluted in the morning to OD_600_ 0.5 then grown in YPD for an additional 4 h followed by dilution to OD_600_ 0.1 (*S. cerevisiae*) or 0.01 (*Candida* spp.), before use as experimental cell suspensions in assays. For filamentous fungi, spores were inoculated from PDA plates into ACM broth at 10^5^ spores mL^–1^ (*A. fumigatus*) or into PDB at 10^4^ spores mL^–1^ (*Z. tritici* and *B. cinerea*). Aliquots (50 μL) of these cell or spore suspensions were transferred to flat-bottom 96-well microtiter plates (Greiner Bio-One; Stonehouse, United Kingdom) with compounds added to specified final concentrations by two-fold serial dilution. The inoculated plates were incubated statically for 24 h at 30°C for *S. cerevisiae* and *Z. bailli*, 37°C for *Candida* spp., 48 h at 37°C for *A. fumigatus* and 7-days at room temperature for *Z. tritici* and *B. cinerea.* Subsequently, OD_600_ was determined with a BioTek EL800 microplate spectrophotometer. Fractional inhibitory concentration (FIC) indices were used to assess potential synergy from the checkerboard results, calculated as: [(compound 1 MIC in combination)/(compound 1 MIC alone)] + [(compound 2 MIC in combination)/(compound 2 MIC alone)] (Hsieh et al., 1993). For continuous growth measurements in the presence of NPs, broth cultures of *S. cerevisiae* were cultivated in 96-well microplates within a BioTek Powerwave XS microplate spectrophotometer (Vallières et al., 2018), in YP (2% peptone, 1% yeast extract) broth supplemented with either 2% D-glucose, glycerol, or ethanol. The growth was monitored from a starting OD_600_ ∼0.1 with continuous shaking at 30°C for 24 h, with OD_600_ readings taken every 30 min.

### Biofilm Inhibition Assay

Biofilm metabolic activity was measured using the XTT (tetrazolium salt, 2,3-bis[2-methyloxy-4nitro-5-sulfophenyl]-2H-tetrazolium-5-carboxanilide) (Sigma, United Kingdom) reduction assay and performed as described previously (Vallières et al., 2020b). Briefly, overnight *C. albicans* cultures were diluted to OD_600_ ∼0.01 in RPMI 1640 medium and 100 μL aliquots transferred to 96-well microtiter plates (Greiner Bio-One) and placed at 37°C. After 2 h non-adherent cells were removed by washing with PBS and plates were incubated at 37°C for 24 h in fresh medium. Biofilms were then washed again with PBS and eugenol and berberine added at specified concentrations or omitted for controls. Cultures were incubated for a further 24 h, then the biofilm was washed and the XTT reaction was performed using 210 μg mL^–1^ XTT and 4.2 μM menadione. Biofilm metabolic activity was measured after 3 h incubation at 490 nm using a BioTek El800 microplate spectrophotometer. The assay was performed in biological triplicate. “Expected values” for combinations were calculated by multiplication of the % biofilm activity determinations for the corresponding individual-compound effects; these were compared with experimental values obtained for the combinations, with statistical analysis by paired *t*-test.

### Establishment of Sub-Inhibitory Concentrations

For the initial determination of sub-inhibitory concentrations (SIC) of selected test compounds (for subsequent use in the high throughput screen), experimental cell suspensions of *S. cerevisiae* W303 in YPD were prepared from overnight cultures as described above. Aliquots (50 μL at OD_600_ 0.2) were mixed with 50 μL of YPD containing the relevant NP, from 2× solutions of the test compounds. In all conditions including solvent-matched controls, solvent concentrations were <1% of the final assay volume. Subsequent growth was measured by OD_600_ determination with a BioTek EL800 microplate spectrophotometer, after 24 h static incubation at 30°C.

### High-Throughput Screening

For high-throughput screens, the four test compounds at their SIC (750 μM eugenol, 12.5 μM β-escin, 350 μM berberine, 50 μM curcumin) were assayed in pairwise combinations against the Puretitre natural compound library (Caithness Biotechnologies, United Kingdom), comprising 200 chemicals at 10 mM, dissolved in DMSO. For the screens, aliquots (1 μL) of each library compound were combined with 49 μL YPD and added to 96-well microtiter plates (Greiner Bio-One). Aliquots (50 μL) of yeast cell suspension (prepared as described above) containing one of the four test compounds (added at double the final desired SIC concentration, see above) were added to the 50 μL library-compound preparations in the microtiter plates. This gave final concentrations of 100 μM of each library compound in 100 μL total per well. Solvent-matched controls at 0.35% DMSO or 0.3% ethanol (70%) were used for control assays without added compounds. Subsequent growth was measured according to OD_600_ determinations with a BioTek EL800 microplate spectrophotometer after 12 h and 24 h static incubation at 30°C. OD_600_ from growth with added compounds was expressed as a percentage of control growth without the compounds. Effect strength [(% growth with library compound) - (% growth with library compound + test compound)] was calculated for each combination; screen “hits” were considered as those combinations showing an effect strength > 50, as described previously (Vallières et al., 2020b). Screens were performed in duplicate.

### Mitochondrial-Membrane Depolarization Assay

Depolarization of the mitochondrial membrane in yeast cells was determined according to rhodamine 123 dye retention using a method adapted from previous reports (da Silva et al., 2016; Alves et al., 2017). After 24 h of exposure to berberine and/or eugenol in checkerboard format, yeast cell suspensions were removed from 96-well plates, spun down in 1.5 mL microcentrifuge tubes at 1,900 × *g* for 3.5 min, then washed with and resuspended in 250 μL phosphate-buffered saline (PBS) before supplementation with a final concentration of 20 μg mL^–1^ rhodamine 123 (Sigma, United Kingdom) and incubation for 30 min at 30°C in the dark. After incubation, cells were washed once with PBS and resuspended in 500 μL PBS then transferred to 5 mL falcon tubes [Becton Dickinson (BD), United Kingdom]. A BD FACS Canto A flow cytometer (blue filter; excitation at 488 nm, emission at 530 nm) was used to determine the fluorescence intensity of cells. A total of 20,000 cells were evaluated per sample and each condition was assessed in technical triplicate for each independent experiment (*n* = 4). Cellular debris was gated out from the analysis using Kaluza software. Median fluorescence intensity (MFI) values for cells treated with NP compounds were transformed to percentages relative to the MFI for minus-compound control cells. “Expected values” for % MFI and statistical comparison with experimental values were calculated as described above for biofilm inhibition.

### Fluorescence Microscopy

Visualization of *S. cerevisiae* cells was performed after 24 h treatment with the EUG + BER combination, rhodamine-123 staining, and washing as described above. Cells were resuspended in 50 μL PBS before mounting. A GXML3201LED fluorescence microscope equipped with a GX-CAM controlled by GXCapture software (GX microscopes, Stansfield, United Kingdom) was used to collect images *via* the FITC filter (excitation, 495 nm; emission, 519 nm) using a ×40 objective lens.

## Data Availability Statement

The original contributions presented in the study are included in the article/Supplementary Material, further inquiries can be directed to the corresponding author.

## Author Contributions

CA designed and performed the experiments, carried out data analysis, and drafted the manuscript. SA conceived the project and contributed to data interpretation and manuscript preparation. Both authors contributed to the article and approved the submitted version.

## Conflict of Interest

The authors declare that the research was conducted in the absence of any commercial or financial relationships that could be construed as a potential conflict of interest.

## Publisher’s Note

All claims expressed in this article are solely those of the authors and do not necessarily represent those of their affiliated organizations, or those of the publisher, the editors and the reviewers. Any product that may be evaluated in this article, or claim that may be made by its manufacturer, is not guaranteed or endorsed by the publisher.

## Footnotes

1
http://www.caithnessbiotechnologies.com/puretitre.html
