# Supplementary material for: Discovery of Natural Products With Antifungal Potential Through Combinatorial Synergy
Source: Front Microbiol. 2022 Apr 26;13:866840. doi: 10.3389/fmicb.2022.866840 (PMC9087349; doi:10.3389/fmicb.2022.866840)
Supplement: Supplementary file 1 [file Data_Sheet_1.pdf]

# SUPPLEMENTARY DATA

**C. R. Augostine and S. V. Avery**

Discovery of Natural Products with Antifungal Potential through Combinatorial Synergy

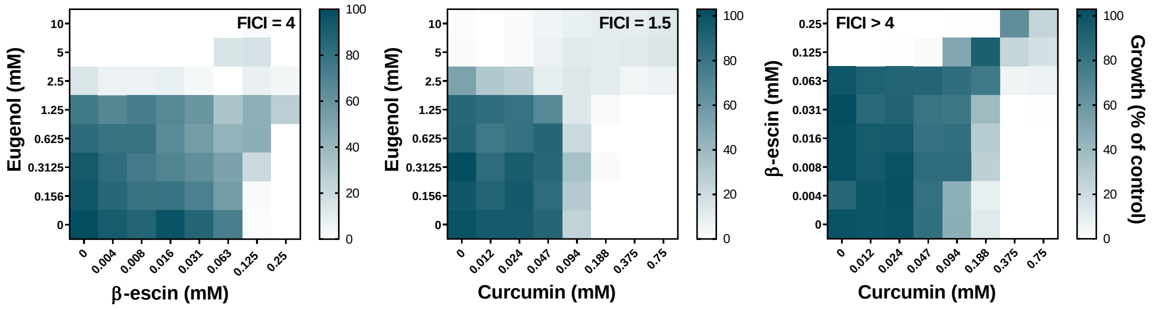


Figure S1. Checkerboard assays of combinatorial growth-effects of eugenol, β-escin and curcumin in *S. cerevisiae* BY4743. Assays were performed according to EUCAST procedure in YPD broth with *S. cerevisiae* BY4743 at the indicated concentrations of eugenol, ß-escin and curcumin. Growth values (scale to the right) represent means from three independent experiments, calculated as percentages of growth ($OD_{600}$) with the NPs relative to the minus-NP control. FICI, fractional inhibitory concentration index, calculated from data after 24 h growth at 30°C; growth values < 5% were assigned as no-growth.[20] Corresponding data for *S. cerevisiae* W303 are shown in Fig. 1.

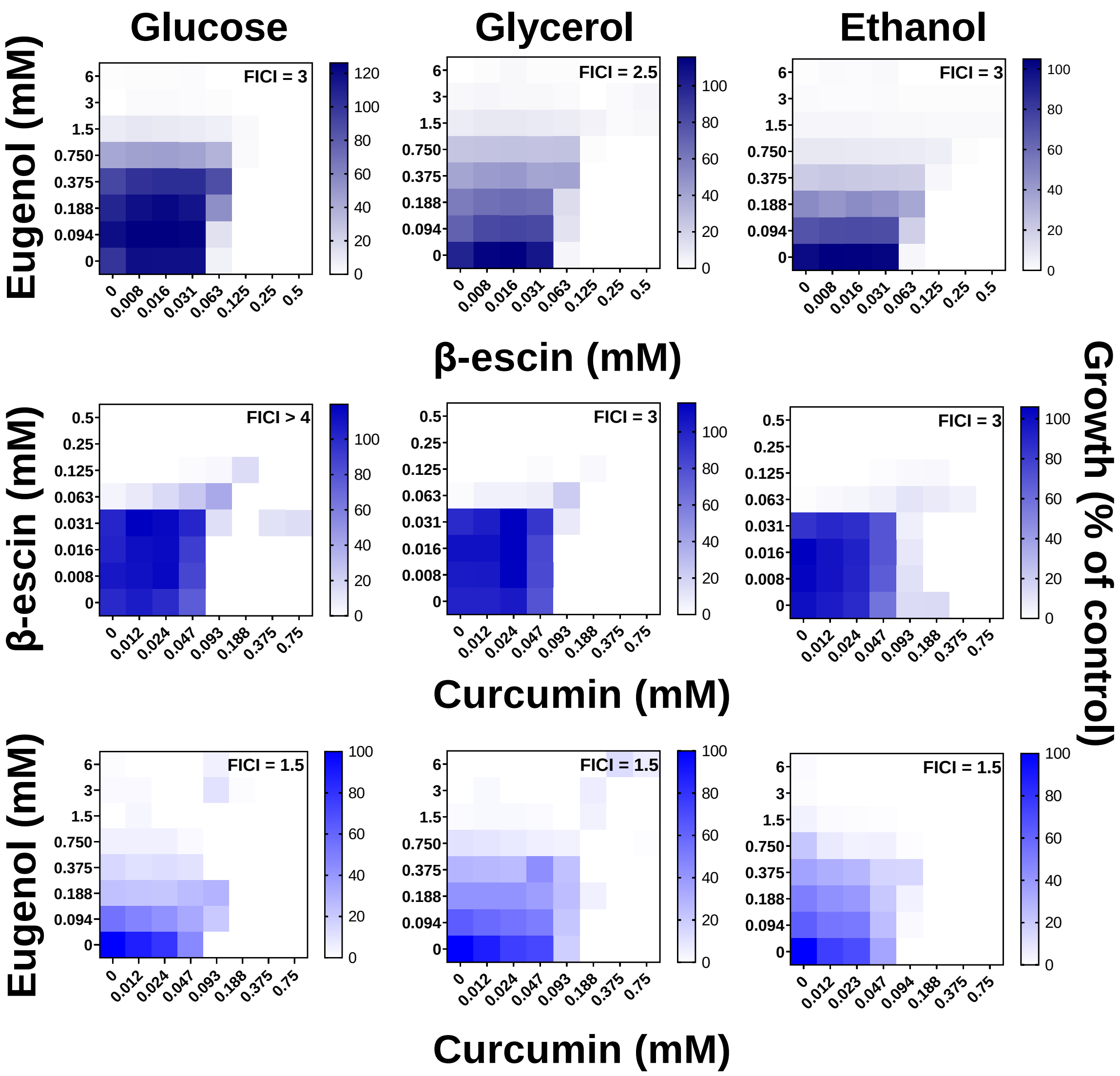


Figure S2. Checkerboard assays of combinatorial growth-effects performed according to EUCAST procedure in YP broth (+2% either glucose, glycerol or ethanol) with *S. cerevisiae* W303 at the indicated concentrations of eugenol, ß-escin and curcumin. The growth values represent the mean of three independent experiments calculated as percentages of growth ($OD_{600}$) with the natural products relative to the minus-NP control. FICI, fractional inhibitory concentration index, calculated from the data after 24 h growth at 30°C and where growth < 5% of the control was assigned as no-growth.[20]

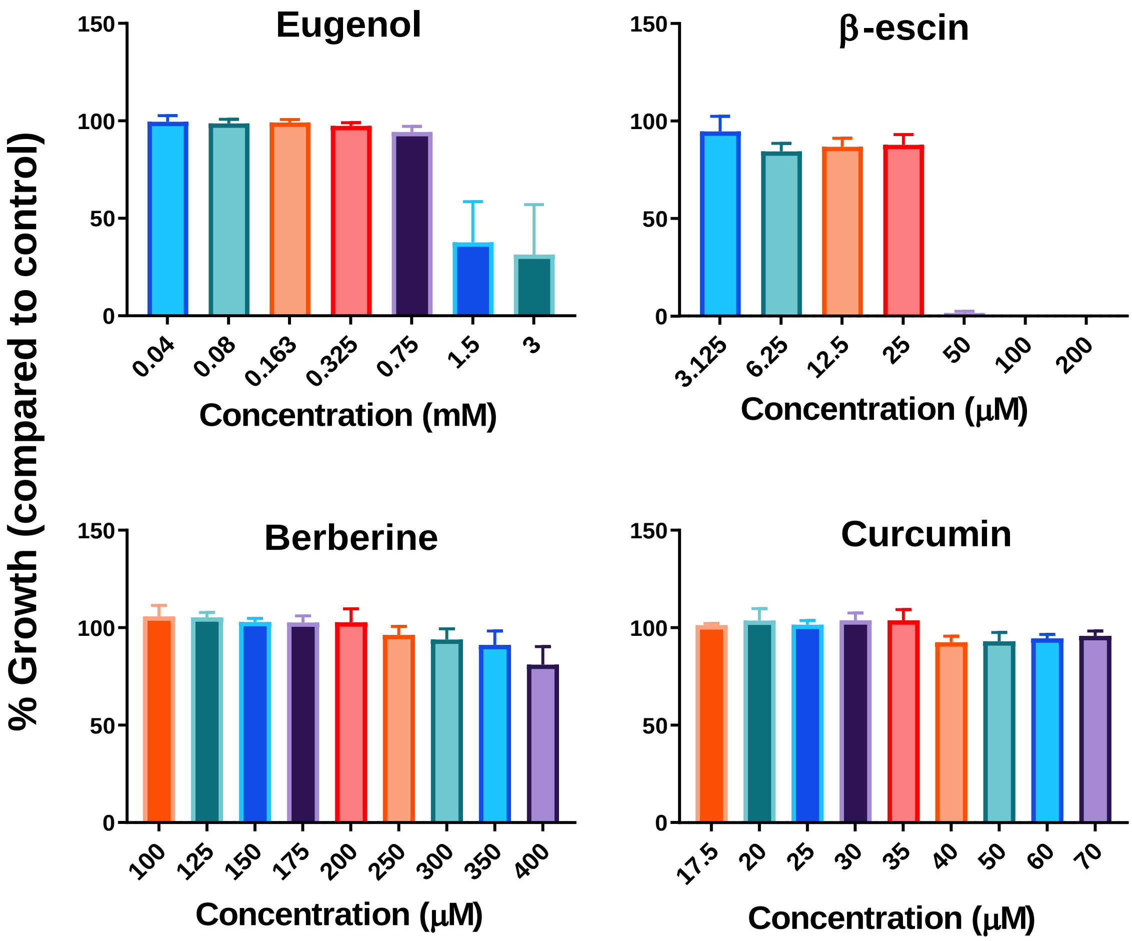


Figure S3. Characterisation of sub inhibitory concentrations (SIC) of selected NPs in *S. cerevisiae* W303. Growth was assessed ($OD_{600}$) after 24 h incubation in YPD broth with the indicated concentrations of eugenol, curcumin, β-escin or berberine. Percentage growth was calculated by comparison with $OD_{600}$ obtained for solvent-only, no-drug controls. Mean values + SEM from three independent experiments are plotted.

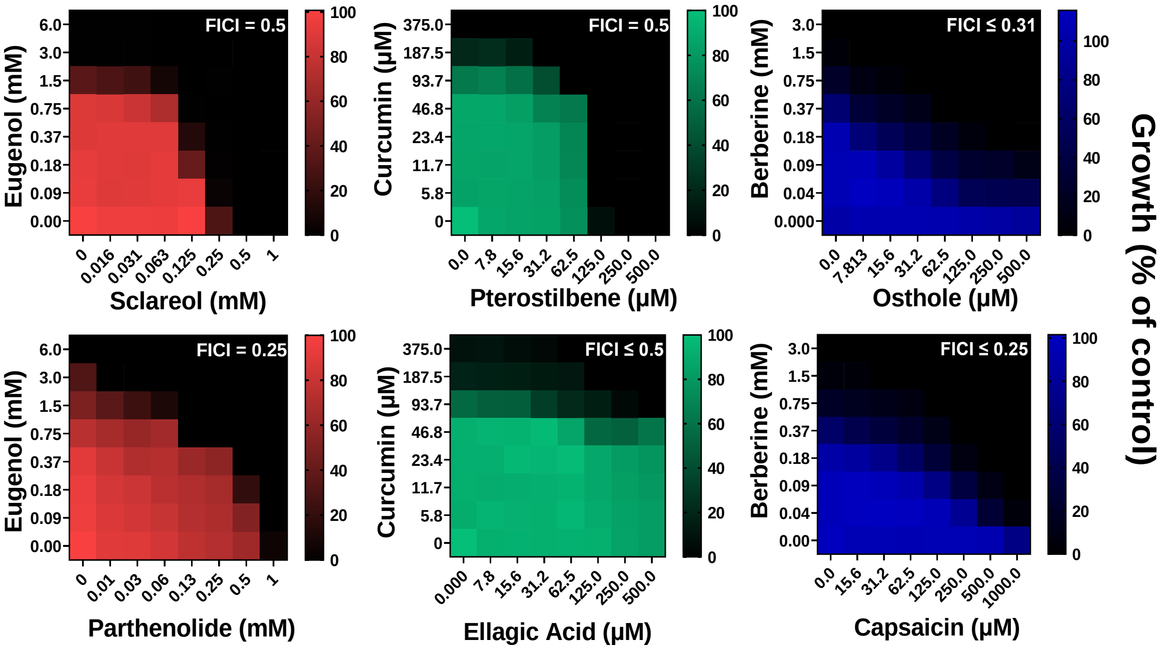


Figure S4. Checkerboard corroboration in *S. cerevisiae* W303 of additional synergies arising from the prior screen. Checkerboard assays of combinatorial growth-effects performed according to EUCAST procedure in YPD broth with *S. cerevisiae* W303 at the specified concentrations of the indicated natural products. The growth values represent the mean of three independent experiments calculated as percentages of growth ($OD_{600}$) with the natural products relative to the minus-NP control, after 24 h growth at 30°C. FICI, fractional inhibitory concentration index, where growth < 5% of the control was assigned as no-growth.[20]

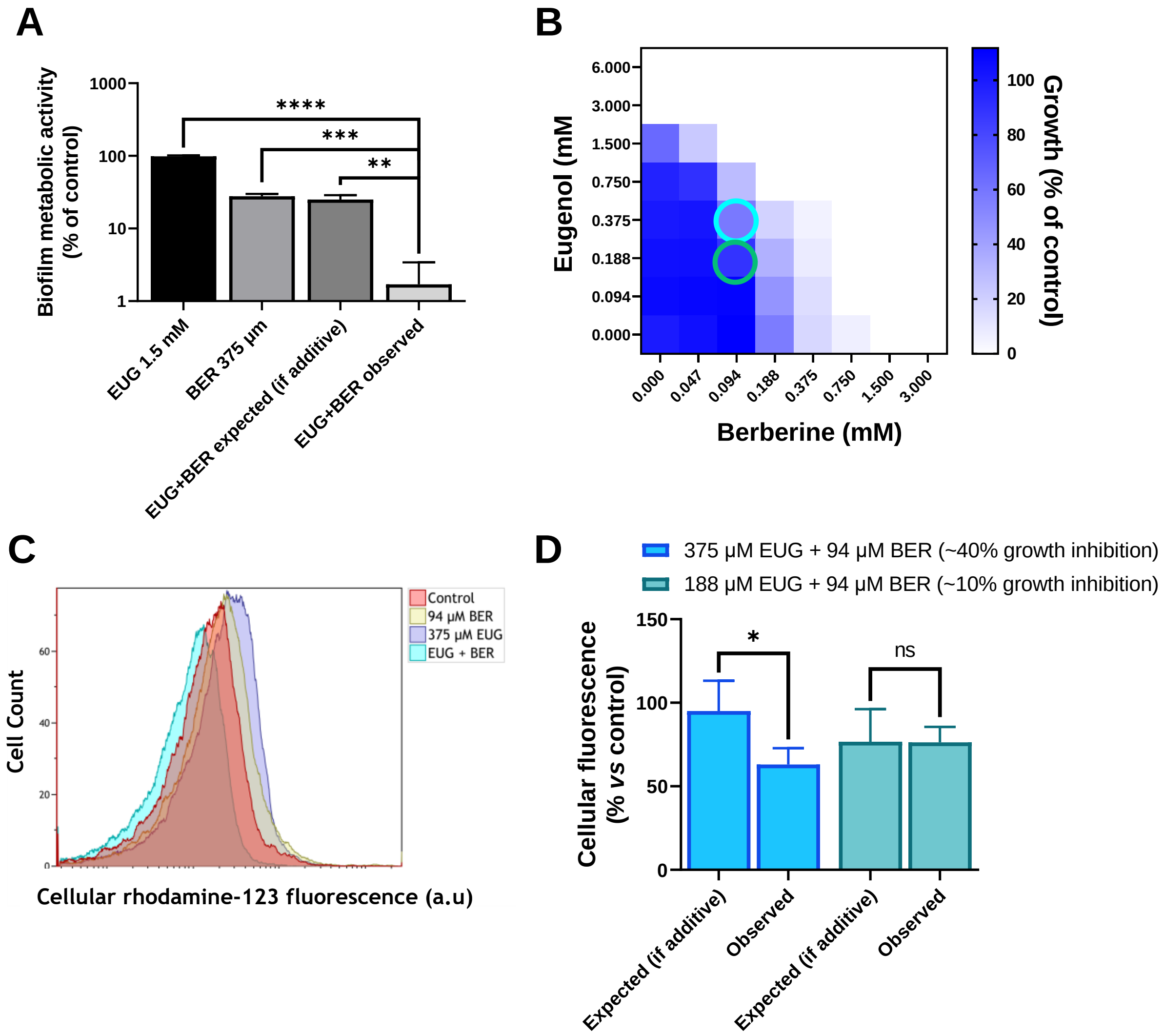


Figure S5. Biofilm metabolic activity and mitochondrial membrane depolarization in eugenol- and berberine-treated *C. albicans* cells. (A) Activity of EUG + BER was assessed against 24 h old biofilms, when EUG + BER were added at the specified concentrations and incubated for a further 24 h before assay of metabolic activity by XTT reduction (see Methods). Data are plotted as a percentage of the solvent only (NP-free) control. Values represent means +SEM from three independent experiments: **, p <0.01; ***, p <0.001; ****, p <0.0001 according to unpaired t-tests. (B) Checkerboard assays of combinatorial growth-effects were performed as described in Figures 1 and 4; combination concentrations are circled where subsequently tested for mitochondrial membrane depolarization (C,D). (C) Flow cytometric histograms for cells incubated for 24 h without (control) or with the indicated concentrations of EUG and BER and stained with rhodamine 123; a.u., arbitrary units. (D) Observed effects of combinations were obtained experimentally from median fluorescence of rhodamine 123-stained cells exposed to the EUG + BER combination (derived from corresponding flow cytometric data as in B), normalized to the no drug control (100%). Expected effects were calculated by multiplication of the two % median-fluorescence determinations obtained for the corresponding individual-compound effects. Values represent means +SEM from three independent experiments: *, p<0.05 according to paired, one-tailed t-test. EUG, Eugenol; BER, Berberine.

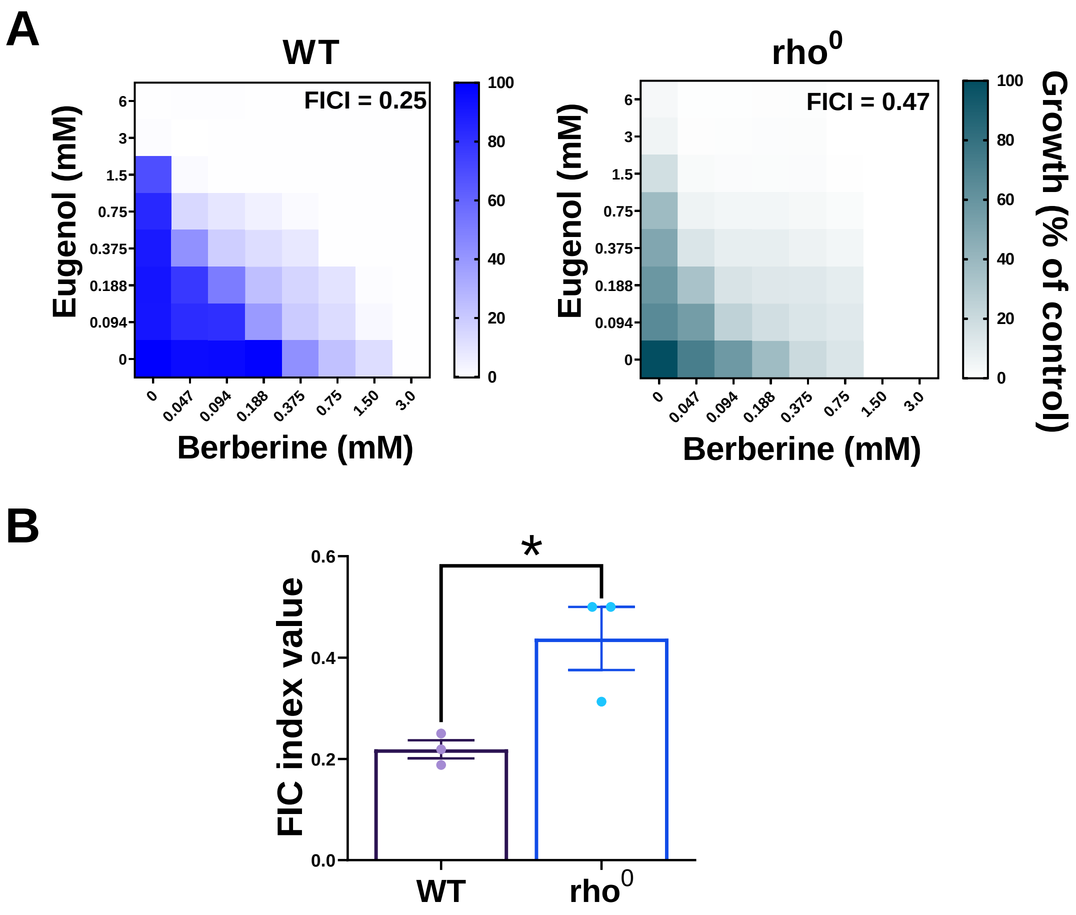


Figure S6. Checkerboard assessment of EUG + BER synergy in wild type and rho$^0$ *S. cerevisiae*. (A) Checkerboard assays of combinatorial growth-effects performed according to EUCAST procedure in YPD broth with *S. cerevisiae* W303 (WT) and a derived rho$^0$ mutant at the indicated concentrations of eugenol and berberine. The growth values represent the mean of three independent experiments calculated as percentages of growth ($OD_{600}$) with the natural products relative to the minus-NP control, after 24 h growth at 30°C. FICI, fractional inhibitory concentration index, calculated from the data and where growth < 5% of the control was assigned as no-growth.[20] (B) Fractional inhibitory concentration indices determined from three independent checkerboard experiments, with bar-height showing mean ±SEM. *, $p < 0.05$, according to unpaired t-test.

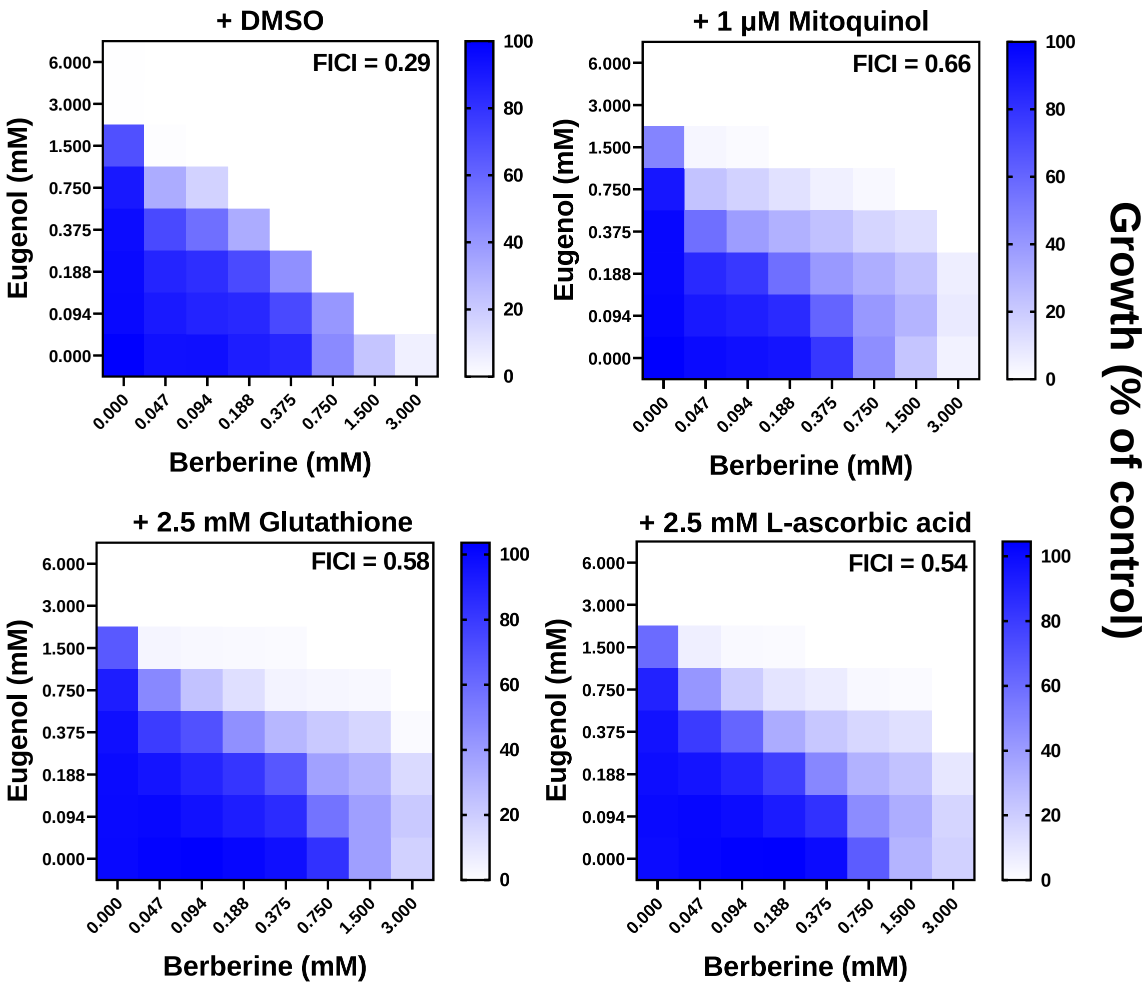


Figure S7. Checkerboard assessment of EUG + BER synergy with or without the addition of different antioxidants. Checkerboard assays of combinatorial growth-effects performed according to EUCAST procedure in YPD broth with *S. cerevisiae* W303 at the indicated concentrations of eugenol and berberine with the inclusion of the indicated antioxidant concentrations. The growth values represent the mean of three independent experiments calculated as percentages of growth ($OD_{600}$) with the natural products relative to the minus-NP control, after 24 h growth at 30°C. FICI, fractional inhibitory concentration index values were calculated from the data and where growth < 5% of the control was assigned as no-growth.[20]
